# Changes in the transcriptome of morula-stage bovine embryos caused by heat shock: relationship to developmental acquisition of thermotolerance

**DOI:** 10.1186/1477-7827-11-3

**Published:** 2013-01-15

**Authors:** Miki Sakatani, Luciano Bonilla, Kyle B Dobbs, Jeremy Block, Manabu Ozawa, Savita Shanker, JiQiang Yao, Peter J Hansen

**Affiliations:** 1 Kyushu-Okinawa Agricultural Research Center, National Agriculture and Food Research Organization, Kumamoto, 861-1192, Japan; 2 Department of Animal Sciences, D.H. Barron Reproductive and Perinatal Biology Research Program, and Genetics Institute, University of Florida, Gainesville, FL, 32611-0910, USA; 3 Ovatech LLC, Gainesville Florida, FL, 32608, USA; 4 Laboratory of Developmental Genetics, Institute of Medical Science, University of Tokyo, Tokyo, Japan; 5 Interdisciplinary Center for Biotechnology Research, University of Florida, Gainesville, FL, 32610, USA; 6 Present address: Minitube International Center for Biotechnology, Mt. Horeb, WI, 53572, USA

## Abstract

**Background:**

While initially sensitive to heat shock, the bovine embryo gains thermal resistance as it progresses through development so that physiological heat shock has little effect on development to the blastocyst stage by Day 5 after insemination. Here, experiments using 3’ tag digital gene expression (3’DGE) and real-time PCR were conducted to determine changes in the transcriptome of morula-stage bovine embryos in response to heat shock (40 degrees C for 8 h) that could be associated with thermotolerance.

**Results:**

Using 3’DGE, expression of 173 genes were modified by heat shock, with 94 genes upregulated by heat shock and 79 genes downregulated by heat shock. A total of 38 differentially-regulated genes were associated with the ubiquitin protein, UBC. Heat shock increased expression of one heat shock protein gene, *HSPB11,* and one heat shock protein binding protein, *HSPBP1*, tended to increase expression of *HSPA1A* and *HSPB1,* but did not affect expression of 64 other genes encoding heat shock proteins, heat shock transcription factors or proteins interacting with heat shock proteins. Moreover, heat shock increased expression of five genes associated with oxidative stress *(AKR7A2, CBR1, GGH, GSTA4,* and *MAP2K5),* decreased expression of *HIF3A,* but did not affect expression of 42 other genes related to free radical metabolism. Heat shock also had little effect on genes involved in embryonic development. Effects of heat shock for 2, 4 and 8 h on selected heat shock protein and antioxidant genes were also evaluated by real-time PCR. Heat shock increased steady-state amounts of mRNA for *HSPA1A* (P<0.05) and tended to increase expression of *HSP90AA1* (P<0.07) but had no effect on expression of *SOD1* or *CAT*.

**Conclusions:**

Changes in the transcriptome of the heat-shocked bovine morula indicate that the embryo is largely resistant to effects of heat shock. As a result, transcription of genes involved in thermal protection is muted and there is little disruption of gene networks involved in embryonic development. It is likely that the increased resistance of morula-stage embryos to heat shock as compared to embryos at earlier stages of development is due in part to developmental acquisition of mechanisms to prevent accumulation of denatured proteins and free radical damage.

## Background

The newly-formed preimplantation embryo is very sensitive to disruption of its developmental program by heat shock. In the mouse [1] and cow [2], exposure to a temperature that is only 1.5-2.0°C above normal body temperature can disrupt development of 1-cell embryos. While initially sensitive to heat shock, the embryo gains thermal resistance as it progresses through development. Heat shock at the pronuclear stage reduced development of mouse embryos more than heat shock at the 2-cell stage [1]. In the cow, heat shock was more detrimental to development when applied at the 8-cell stage or earlier than when applied later in development [3-6]. Development of bovine zygotes was reduced by a heat shock of 40°C [2]. Moreover, adverse effects of exposure of females to heat stress on embryonic survival become reduced after a few days post-fertilization in sheep [7], pigs [8] and cattle [9].

Two phenomena appear important for developmental acquisition of thermotolerance in the preimplantation embryo. The first is a change in oxidative stress. Free radical production is an important cause of reduced development in heat-shocked embryos; effects of heat shock in vitro [10-13] and heat stress in vivo [14,15] can be reduced in some cases (although not all) [16,17] by administration of antioxidants. In the cow, free radical production increases in response to heat shock when embryos were heat-shocked at Day 0 and 2 relative to fertilization but not when heat shock was imposed at Day 4 or 6 [5]. Amounts of the antioxidant glutathione are also low during early development and do not increase until the 9–16 cell stage [18]. Another reason for the developmental gain in resistance of embryos to heat shock is the activation of the embryonic genome to allow stimulation of cellular pathways that protect cells from stress. In both the mouse and bovine, embryos become more resistant to heat shock at about the same time embryonic genome activation takes place (1–2 cell stage in the mouse [19] and 8–16 cell stage in the cow [20]). The phenomenon of induced thermotolerance, whereby exposure to a mild heat shock induces changes in cellular function to make cells more resistant to a subsequent, more severe heat shock, can occur in bovine blastocysts [21] but not in 2-cell embryos [22]. Much of the change in cellular function leading to thermotolerance involves transcriptional activation of genes for heat shock proteins and other proteins [23].

The purpose of this study was to evaluate changes in the transcriptome of morula-stage bovine embryos in response to heat shock. The goal was to identify genes and gene networks affected by heat shock that would explain the molecular basis for thermal resistance at this stage of development. The transcriptome was assessed using a next-generation sequencing method called 3’ tag digital gene expression (3’DGE). In this procedure, high throughput sequencing is used to read 20–21 bp amplicons of 3’ regions of mRNAs [24,25]. The procedure is particularly useful for cases where mRNA is limited because more gene counts are generated than for procedures in which the whole transcriptome is analyzed [26].

## Methods

### Materials

The fertilization medium SOF-FERT was made by modifying a custom formulation of Synthetic Oviduct Fluid manufactured by Specialty Media (Millipore, Billerica, MA, USA) to create the following formulation: 107.7 mM NaCl, 7.16 mM KCl, 1.19 mM KH_2_PO_4_, 1.17 mM CaCl_2_·H_2_O, 5.3 mM sodium lactate, 0.20 mM sodium pyruvate, 25.07 mM NaHCO_3_, 0.49 mM MgCl_2_·6H_2_O, 6 mg/ml essentially fatty acid free BSA (Sigma, St. Louis, MO, USA), 5 μg/ml gentamicin, 10 μg/ml heparin, and 1.0 mM caffeine. The embryo culture medium was SOF-BE1 and was prepared as described elsewhere [27]. Unless otherwise mentioned, all other chemicals and reagents were purchased from Sigma-Aldrich (St. Louis, MO, USA).

### Production of embryos in vitro

Bovine embryos were produced from slaughterhouse-derived immature oocytes using procedures for in vitro oocyte maturation and fertilization as described previously [2]. Oocytes were inseminated with a pool of sperm from three *Bos taurus* bulls. A different pool was used for each replicate. After 8 h of insemination, cumulus cells were removed from putative zygotes by vortexing and the zygotes were cultured in groups of 30 in 50 μl microdrops of SOF-BE1 covered in mineral oil at 38.5°C and 5% CO_2_ in humidified air. Embryos were cultured until 116 h after insemination (hpi), when they were used for experiments.

### Changes in the transcriptome caused by heat shock as determined by 3’DGE

#### Treatment and RNA purification

At 116 hpi, embryos were either maintained at 38.5°C (control) or moved to an incubator at 40°C (heat shock) and 5% CO_2_ in humidified air for 8 h. Morulae (defined here as embryos > 16 cells) were collected and the zona pellucidae removed by incubation with 0.1% (w/v) protease from *Streptomyces griseus* in Dulbecco’s phosphate buffered saline (DPBS) containing 0.2% (w/v) polyvinylpyrrolidone (PVP). Embryos were then washed with DPBS-PVP, transferred in groups of 50 embryos to a 1.5 mL tube containing 50 μL extraction buffer from the PicoPure RNA isolation kit (Applied Biosystems, Carlsbad, CA, USA), incubated at 42°C for 30 min, and centrifuged at 3,000 g for 2 min. The supernatant fractions were used to prepare total RNA using the PicoPure RNA isolation kit. DNA was digested using the RNase-Free DNase Set (Qiagen, Valencia, CA, USA). The concentration and quality of total RNA were assessed by a Nanodrop ND-1000 (ThermoScientific, Wilmington, DE, USA) and Agilent 2000 Bioanalyzer (Agilent Technologies, Santa Clara, CA, USA). Samples were only used for 3’DGE if RNA integrity was > 8.0. A total of three replicates of 50 morulae each that met this criterion were obtained for each treatment.

#### RNA amplification and 3’-DGE sequencing

Total RNA was processed for cDNA synthesis using the Ovation 3’-DGE system (NuGEN Technologies, Inc., San Carlos, CA, USA). The 3’-DGE libraries were constructed from the resulting double stranded cDNA using the TruSeq™ DNA library preparation kit according to the manufacturer’s instructions (Illumina, San Diego, CA, USA). In brief, 500 ng cDNA was sheared and pooled with 500 ng of un-sheared cDNA and end-repaired by enzymatic polishing with T4 DNA polymerase and E.*coli* DNA polymerase 1 Klenow fragment. A single ‘A’ base was added (A tailing) to the 3’end of the repaired fragments. Illumina pair-end adaptors, essentially consisting of the sequencing primer-annealing sequences, were then ligated to the A-tailed fragments via a 3’ thymine overhang followed by purification using Agentcourt AmpureXP beads (Beckman Coulter, Inc.). The purified ligated DNA was subjected to 11 cycles of PCR amplification to enrich the adaptor modified cDNA libraries using primers complementary to the ends of the adaptors [PCR primers PE 1.0 and PCR primer PE 2.0 (Illumina)]. The purified ligated population was resolved on a 2% (w/v) TAE-agarose gel and 300–500 bp fragments were excised and purified using Qiaquick gel extraction kit. The amplified libraries were quantified using the Agilent DNA high-sensitivity kit on an Agilent 2100 Bioanalyzer (Agilent Technologies, Santa Clara CA, USA). Based on the calculated values, the barcoded libraries were pooled in equal concentration for cluster generation and sequencing onto two lanes of Illumina GAIIx Genome Analyzer (Illumina, San Diego, CA, USA). Cluster formation, primer hybridization and sequencing runs were done according to manufacturer’s protocols. We ran one lane for single-end 101 cycles and a second lane for paired-end 2x101 cycles. Image analysis and base calling were performed using the Illumina Pipeline, where sequence tags were obtained after purity filtering.

#### Data analysis

For each treatment, there were a total of three replicates or 6 sequencing data files in total. A technical replication was performed on one control sample and one heat shock sample. Technical replicates were merged as one replicate for further analysis.

Raw sequencing reads were initially processed with GenomeQuest tools [28]. Ambiguous residues were trimmed off from both sides of the sequence. Bases with Phred quality below 12 from the 3’ end of the sequence were removed. Reads that were shorter than 40 bases or that contained more than 10 bases with quality below 12 were discarded. Also excluded were reads consisting of repetitive single bases for more than 60% of the length at the 3’ end. A total of 70-84% of reads were retained after the cleanup step.

For mapping of reads, *B. taurus* genomic sequences *bosTau6* (repeat masked) were downloaded from the UCSC genome browser [29]. Sequencing reads of each sample were mapped independently to the reference sequences using Tophat 1.4.1 (University of Maryland) [30]. Tophat splits reads to segments and joins segment alignments. A maximum of one mismatch in each of the 25 bp segments was allowed. The unmapped reads were collected and mapped to the reference using Bowtie 0.12.8 [31] allowing two mismatches. In the third step, the unmapped reads were further mapped to the mRNA sequences using novoalign 2.08.02 [32] allowing for three mismatches for each read. The mRNA sequences of *B. taurus* were downloaded from Ensemble [33]. A total of 22,915 genes were obtained.

The number of mapped reads for each individual gene was counted by using HTSeq tool [34] with intersection-nonempty mode. HTSeq takes two input files - the bam or sam files of mapped reads and a gene model file. Ensemble gene annotation file in GTF format was downloaded from Ensemble genome browser. Three criteria were used to identify a gene as being regulated by heat shock: average count of > 2 in at least one treatment, treatment effect of P<0.05 (paired Student *t*-test), and a fold change (heat shock/control) of > 1.5 or < 0.67.

#### Analysis of gene networks and functions

Differentially expressed genes were analyzed for functional and regulatory relationships using Ingenuity Pathways Analysis (IPA; Build 172788; release date Aug 11, 2012). The reference set was the Ingenuity Knowledge Base (genes only) and both direct and indirect relationships that were experimentally observed were included. A total of 151 of the 173 differentially expressed genes was present in the reference database. A P value of 0.05 or less was considered significant when determining pathways and functions in which differentially expressed genes were overexpressed.

The list of differentially expressed genes was also annotated by the Database for Annotation, Visualization and Integrated Discovery (DAVID; (DAVID Bioinformatics Resources 6.7, National Institute of Allergy and Infectious Diseases [35]). A total of 116 genes were included in the analysis of gene functional classification, ontology and pathway analysis.

### Temporal changes in expression of selected genes in response to heat shock

At 116 hpi, embryos were either maintained at 38.5°C or cultured at 40°C and 5% CO_2_ in humidified air. Morula-stage embryos (n=10) were harvested from microdrops at 2, 4, or 8 h (different drops used for each time point). The experiment was replicated eight times so that there were eight pools of 10 embryos each for each time-treatment combination.

RNA was extracted as follow. The zona pellucida was removed as described previously. Embryos were washed with DPBS-PVP and RNA purified as described earlier except the source of DNAse was New England Biolabs (Ipswich, MA, USA).

RNA was reverse transcribed using a commercial kit (High Capacity cDNA Reverse Transcription Kit, Applied Biosystems). Conditions were 25°C for 10 min, 37°C for 120 min and 85°C for 5 min. The cDNA was subjected to real-time PCR to determine expression of *HSPA1A*, *HSP90AA1, SOD1*, and *CAT* using a CFX96 Real-Time System (Bio-Rad, Hercules, CA), the SsoFast Eva-green Supermix reagent (Bio-Rad) and previously-described primers [2]. Briefly, the reaction mixture consisted of 10 μL of SsoFast EvaGreen Supermix, 7.2 μL of DEPC water with template cDNA (2 μL, 0.5 embryo equivalents) and 0.4 μL each of forward and reverse primer (final concentration of each = 0.2 μM). Amplification reactions were performed in replicates of 5 at 95°C for 30 sec followed by 40 cycles of 95°C for 5 sec and 60°C for 10 sec. A negative control for each sample was included that consisted of amplification without reverse transcriptase. C_t_ values of each gene were calculated by Bio-Rad CFX Manager ver. 1.6 (Bio-Rad). Gene expression values were normalized by the C_t_ value of the reference gene *GAPDH.* Fold changes in expression were calculated relative to values for embryos cultured at 38.5°C for 2 h using the ΔΔC_t_ method.

Gene expression data were analyzed by least-squares analysis of variance using the GLM procedure of the Statistical Analysis System (version 9.2 TA Level 2M2, SAS Institute, Cary, NC, USA). Replicate was considered a random effect while temperature and time were considered as fixed. Tests of significance were performed using appropriate error terms. Data analyzed were ΔΔCt values and results are graphed as fold-change differences. Results are shown as least squares means ± SEM.

### Disruption in development of morulae by heat shock

At 116 hpi, microdrops of embryos were either maintained at 38.5°C or were moved to a new incubator and exposed to heat shock at 40°C and 5% CO_2_ in humidified air for 8 h. Afterwards, heat-shocked embryos were returned to 38.5°C and 5% CO_2_ in humidified air and cultured through day 8 (day 0 = insemination) when blastocyst formation was determined and blastocysts were harvested for determination of numbers of inner cell mass (ICM) and trophectoderm (TE) cells by differential staining [2]. The experiment was replicated five times. Data were analyzed by least-squares analysis of variance using the GLM procedure of SAS. Replicate was considered a random effect, temperature was considered as fixed and the temperature x replicate interaction was used as the error term for temperature. Results are shown as least squares means ± SEM.

## Results

### Changes in global gene expression caused by heat shock

A total of 173 genes were modified by heat shock, with 94 genes upregulated by heat shock and 79 downregulated. A list of the differentially expressed genes is presented in Additional file 1: Table S1.

Using the IPA procedure of Ingenuity, differentially expressed genes were associated significantly with 22 molecular and cellular functions, with the most significant associations being with cell morphology (19 molecules), cellular assembly and organization (28 molecules), cell death and survival (20 molecules), cell-to-cell signaling and interaction (17 molecules) and lipid metabolism (18 genes).

#### ***Genes involved in cellular stress and survival***

**Figure 1:**
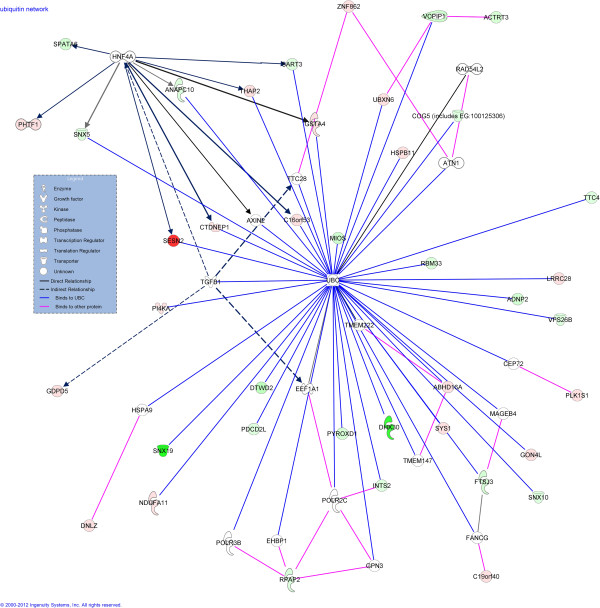
**Genes encoding for proteins that interact with ubiquitin C (UBC) that were increased (red) or decreased (green) in expression by heat shock.** Binding with UBC is indicated by blue lines while binding between other proteins is indicated by pink lines. Other relationships are indicated by arrows. Data were generated using IPA software.

**Figure 2:**
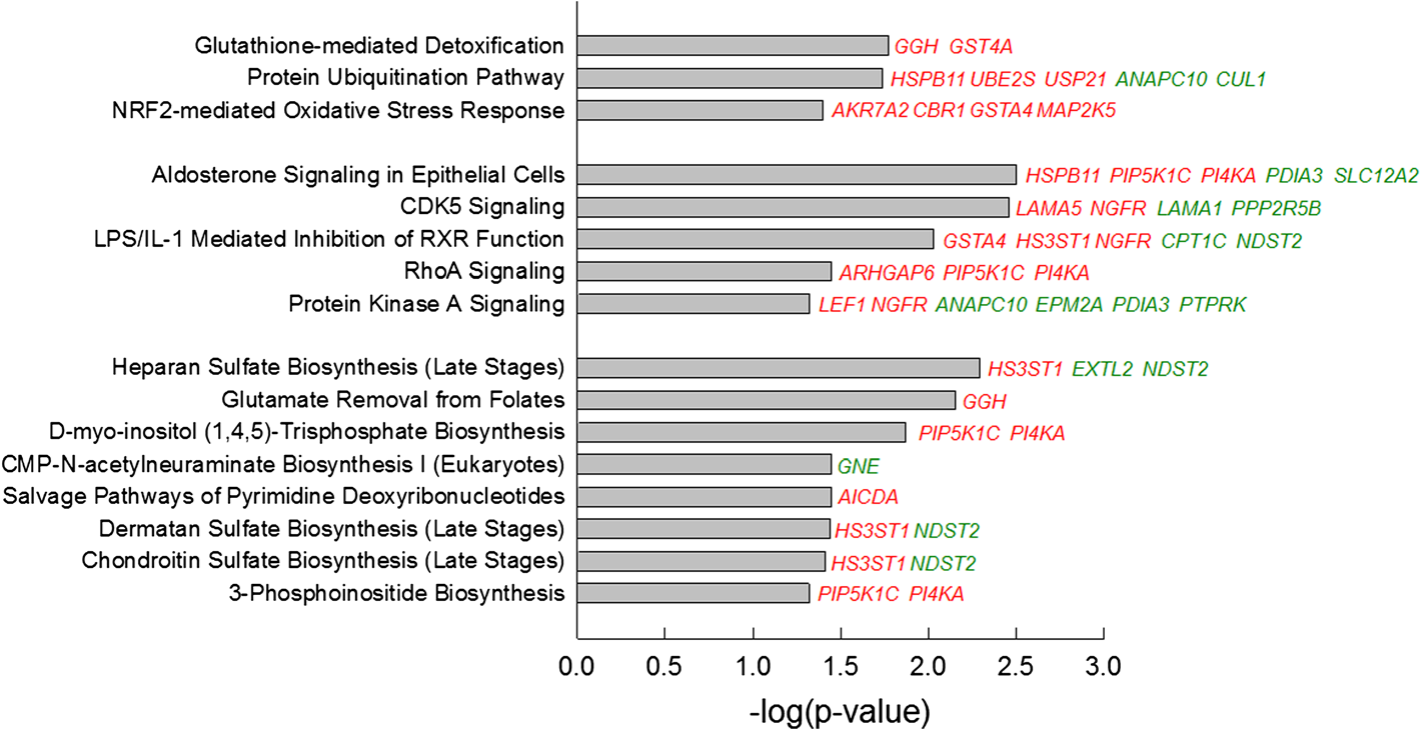
**Canonical pathways in which there was significant overrepresentation of differentially expressed genes.** Data were generated using IPA software. The level of statistical significance is shown on the x-axis. Three clusters of pathways are shown – cellular death and survival (top), cell signaling (middle), and metabolic pathways (bottom). Genes upregulated by heat shock are shown in red and downregulated genes are shown in green.

Gene networks and gene relationships identified using IPA were initially scrutinized for effects of heat shock on genes and gene networks involved in cell death and survival. A total of 38 differentially-regulated genes (25% of the differentially expressed genes available for analysis) were associated with the ubiquitin protein, UBC (Figure 1). These included molecules that bind to UBC (11 genes upregulated by heat shock and 18 downregulated by heat shock) as well as molecules that interact with molecules that bind UBC (6 upregulated and 3 downregulated genes). There were also three canonical pathways related to cell stress and survival identified as having significant representation of differentially expressed genes (Figure 2). These were glutathione-mediated detoxification (two genes upregulated by heat shock), protein ubiquitination pathway (3 upregulated and 2 downregulated) and NRF2-mediated oxidative stress response (4 upregulated genes). Using DAVID, the positive regulation of apoptosis ontology was also found to contain overrepresentation of differentially expressed genes, with *BOK, NGFR, STK4,* and *SERINC3* increased by heat shock and *PDIA3* decreased by heat shock.

Further analysis of 42 genes related to removal of free radicals (peroxidase genes, glutathione metabolism genes, thioredoxin, genes containing thioredoxin domains or involved in thioredoxin metabolism, glutaredoxin genes, superoxide dismutase genes, and catalase) indicated that none were significantly affected by heat shock. For example, the fold-change due to heat shock was 1.3 for *CATA*, 1.2 for *GPX2*, 1.1 for *GLRX1*, 1.0 for *GLRX3*, 1.0 for *SOD1*, 1.0 for *SOD2*, 1.0 for *TXN*, and 1.1 for *TXN2.* Hypoxia-induced factors and mTOR are also regulated by free radicals [36]. There was no significant effect of heat shock on expression of *HIF1A* (0.6 fold-change) or components of the mTOR complex [for example, *MTOR*, 0.8-fold; *RICTOR*, 0.80-fold, and *RPTOR* (1.8-fold)] but *HIF3A* was significantly reduced by heat shock (0.3 fold; P=0.0007).

Expression of heat shock protein genes was not generally affected by heat shock. We looked at expression of 68 genes involved in heat shock protein responses, including heat shock proteins, heat shock transcription factors and heat shock protein binding proteins. Results from a selection of these genes are shown in Table 1. Heat shock increased expression of *HSPB11* (P<0.05) and tended to increase expression of *HSPA1A* (P=0.06) and *HSPB1* (P=0.09). Heat shock also increased (P<0.05) expression of the heat shock binding protein gene, *HSPBP1.* None of the other heat shock protein genes, heat shock transcription factors, or heat shock protein binding proteins was affected by heat shock.

**Table 1:** Effects of heat shock on expression of selected genes involved in the heat shock protein response

**Gene symbol**	**Description**	**Expression level, control**	**Expression level, heat shock**	**Fold change**	**P**
*HSPA1A*	Heat shock 70 kDa protein 1A	3	6	2.11	0.063
*HSPB11*	Heat shock protein family B (small), member 11	26	53	2.0	0.027
*HSPB1*	Heat shock 27kDa protein 1	607	982	1.62	0.085
*HSPH1*	Heat shock 105 kDa/110kDa protein 1	924	1377	1.49	0.208
*HSPBP1*	heat shock 70kDa binding protein, cytoplasmic cochaperone 1	13	20	1.48	0.042
*DNAJC28*	DnaJ (Hsp40) homolog, subfamily C, member 28	9	12	1.30	0.513
*HSP90AA1*	Heat shock protein 90kDa alpha (cytosolic), class A member 1	38026	47565	1.25	0.439
*HSPA8*	heat shock 70kDa protein 8 (hsc70)	2949	3583	1.21	0.471
*HSP90AB1*	Heat shock protein 90kDa alpha (cytosolic), class B member 1	1135	1359	1.2	0.359
*HSPE1*	Heat shock 10kDa protein 1 (chaperonin 10)	57871	60682	1.05	0.796
*HSPD1*	heat shock 60kDa protein 1 (chaperonin)	154	161	1.05	0.869
*HSPB6*	heat shock protein, alpha-crystallin-related, B6	12	12	1.00	1.00
*HSF1*	Heat shock factor protein 1	87	87	1.00	0.962
*DNAJB12*	DnaJ (Hsp40) homolog, subfamily B, member 12	22	18	0.83	0.053
*DNAJC25*	DnaJ (Hsp40) homolog, subfamily C , member 25	150	125	0.83	0.075
*HSF2*	Heat shock factor protein 2	65	48	0.73	0.444

#### Genes important for embryonic development

There were no canonical pathways classified by IPA as being involved in organismal growth and development or classified as involved in cellular growth, proliferation, or development that contained significant overrepresentation of differentially expressed genes.

In addition to pathways already described under cell stress, there were five cell signaling pathways containing differentially expressed genes identified by IPA (Figure 2). The most significant pathway was for aldosterone signaling. Using DAVID, the WNT signaling pathway was also identified as a pathway containing an overrepresentation of differentially expressed genes. In particular, heat shock increased expression of *AXIN1* and *LEF1* and decreased expression of *CUL1* and *PPP2A*.

An additional eight canonical pathways categorized as metabolic pathways by IPA contained significant representation of differentially expressed genes (Figure 2). Three of these pathways involve synthesis of mucopolysaccharides (heparan sulfate, dematan sulfate, and chondroitin sulfate). Another pathway, CMP-N-acetylneuraminate biosynthesis I, contained a gene downregulated by heat shock, *GNE*, that is involved in synthesis of a sialic acid precursor. Two pathways involved in synthesis of phosphatidylinositol 4,5-bisphosphate (D-myo-inositol (1,4,5)-trisphosphate biosynthesis and 3-phosphoinositide biosynthesis) contained two genes upregulated by heat shock (*PIP5K1C, PI4KA*). Glutamate removal from folates was represented because *GGH* was upregulated by heat shock. Salvage pathway of pyrimidine deoxyribonucleotides was represented because heat shock upregulated expression of *AICDA*, which participates in RNA editing by causing deamination of cytidine.

PUBMED was also queried to identify differentially regulated genes that have been implicated in preimplantation development. Only two such genes were identified: *LEF1*, involved in TE differentiation [37], which was increased by heat shock 1.6 fold, and *CUL1*, which is necessary for development in the mouse [38] and was decreased by heat shock (0.58 fold).

#### Putative transcription factors involved in heat-shock induced changes in gene expression

**Figure 3:**
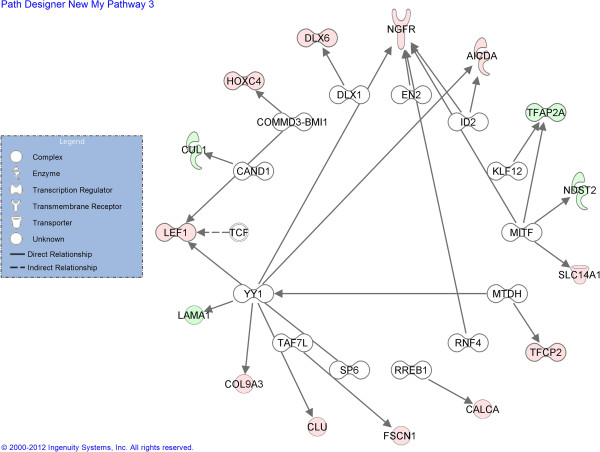
**Transcription factors identified as potential upstream regulators of genes regulated by heat shock.** Data were generated using IPA software. Relationships were P<0.05 in all cases.

Ingenuity was used to identify transcription factors that regulate genes that were regulated in response to heat shock. A total of 14 transcription factors were significant. Together, these control expression of 15 of the differentially-regulated genes (Figure 3). HSF1 or HSF2, which control expression of heat shock protein genes, and NRF2, which controls regulation of antioxidant genes, were not identified.

### Temporal response of gene expression to heat shock as determined by real-time PCR

**Figure 4:**
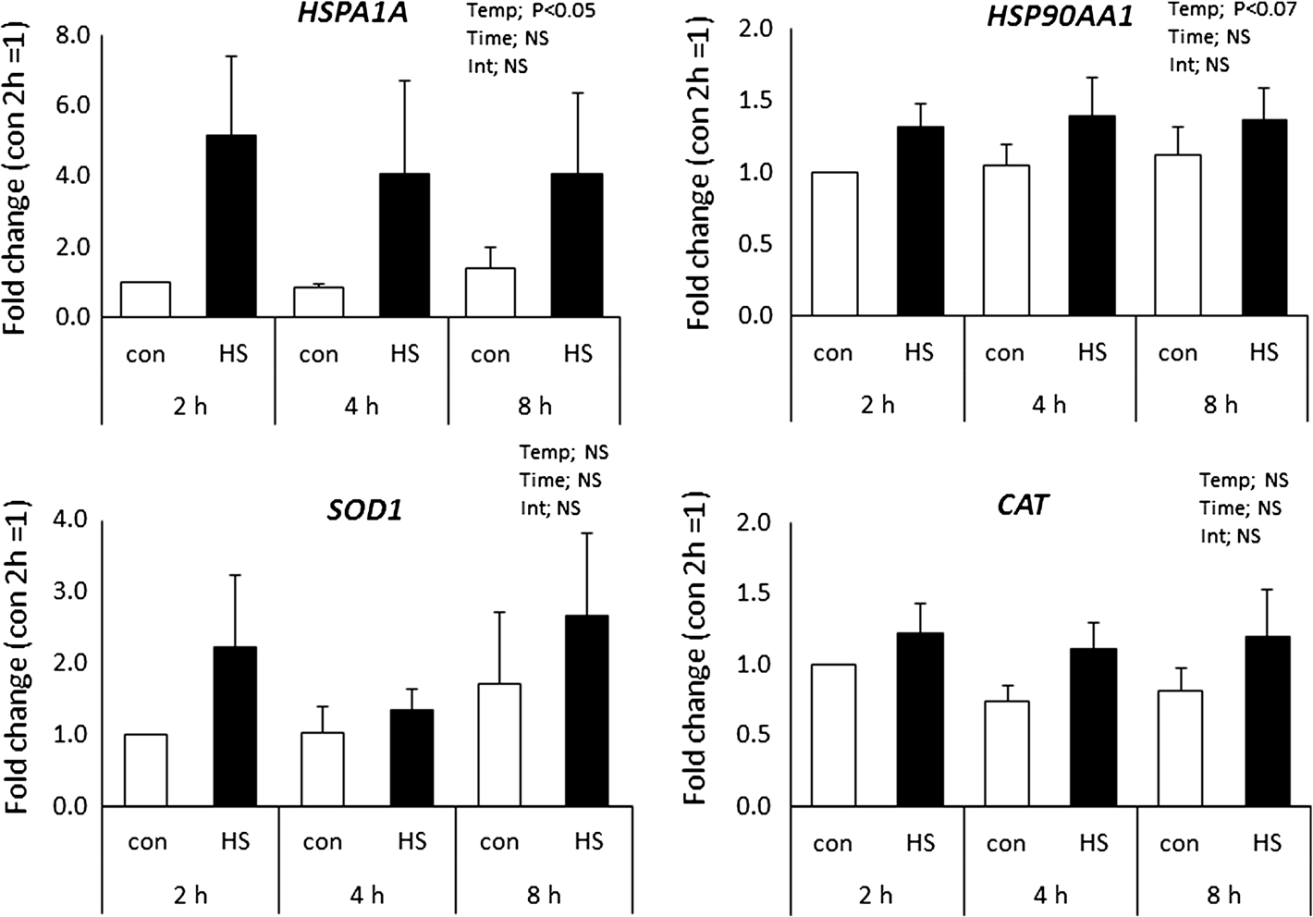
**Changes in expression of selected heat shock protein genes (*HSPA1A *and *HSP90AA1*) and antioxidant genes (*SOD1 *and *CAT*) after culture at 40°C for 2, 4 or 8 h. Con=culture at 38.5°C and HS=culture at 40°C.** Data are least-squares means ± SEM of results from eight replicates. The probability values for effects of temperature (Temp), time and the interaction (Int) are shown in each graph.

Results from 3’DGE indicated that heat shock caused little change in expression of genes for heat shock proteins or antioxidant enzymes, with a tendency for a slight increase in expression of *HSPA1A* and no effect on most heat shock protein genes or antioxidant genes. To confirm these observations and to determine whether responses to heat shock would be different after other times of exposure, the effects of heat shock on steady-state amounts of mRNA for *HSPA1A, HSP90AA, SOD1* and *CAT* after 2, 4 or 8 h of exposure were determined (Figure 4). Heat shock increased steady-state amounts of mRNA for *HSPA1A* (P<0.05) and tended to increase amounts of *HSP90AA1* (P<0.07) but had no effect on expression of *SOD1* or *CAT*. As indicated by the lack of a time x temperature interaction, the increase in mRNA for *HSPA1A* and *HSP90AA1* caused by heat shock occurred after 2 h of heat shock and was similar in magnitude at later times examined.

### Development of heat-shocked embryos to the blastocyst stage

An experiment was conducted to confirm that exposure of morula-stage embryos to a heat shock of the magnitude used for the gene expression studies (40°C for 8 h) would not compromise development to the blastocyst stage. There was no effect of heat shock on the proportion of inseminated oocytes that became blastocysts or on blastocyst cell number (ICM, TE or total) (Table 2).

**Table 2:** Effect of exposure of embryos at 116 h after insemination to 40°C for 8 h on percent of inseminated oocytes that cleaved and that developed to the blastocyst stage and on blastocyst cell number [inner cell mass (ICM), trophectoderm (TE) and total]

**Treatment**	**Number of oocytes (replicates)**	**Percent cleaved**	**Percent developed to blastocyst**	**ICM cell number**	**TE cell number**	**Total**
Control	293 (5)	74.0 ± 3.5	30.4 ± 1.1	28.3 ± 1.4	75.2 ± 4.1	103.5 ± 5.0
Heat shock	320 (5)	74.5 ± 5.3	28.8 ± 5.3	28.1 ± 1.1	79.3 ± 3.9	107.4 ± 4.5

## Discussion

Within the period of a few days, the newly-formed bovine embryo is transformed from an organism that is unable to successfully survive exposure to elevated temperature to one that is capable of continuing development after exposure to physiological heat shock. Thermal tolerance becomes acquired at about the 8–16 cell stage [2-6], which is coincident with the period of development when the embryonic genome becomes fully activated [20]. Here we hypothesized that the morula-stage embryo is resistant to heat shock because it can undergo transcriptional changes in genes related to cellular survival that stabilize cellular function at elevated temperature.

In fact, however, the transcriptional response to heat shock was muted. Only a few genes involved in the heat shock protein response or antioxidant function were increased by heat shock. Thus, it is likely that heat shock at this stage of development does not cause a large increase in the signals leading to transcription of heat shock protein genes (denatured protein, ref. [39]) or antioxidant genes (free radicals, ref [40-42]). Put differently, there was not a large-scale transcriptional response of heat shock protein genes or antioxidant genes in response to heat shock because the increased need for the proteins encoded by these genes is minimal. Embryonic resistance of the morula to physiological heat shock as compared to embryos at earlier, more thermosensitive stages is more likely to be due to possession of mechanisms to prevent accumulation of denatured proteins and free radical damage rather than due to differences in transcriptional regulation of heat shock protein and antioxidant genes. Indeed, the two-cell embryo, which is very sensitive to heat shock is capable of increasing transcription of *HSPA1A*[43] and synthesis of HSP70 [44,45] in response to heat shock. In addition, the steady-state amount of mRNA for *HSPA1A* in the two-cell embryo not exposed to heat shock is higher than amounts in the Day 5 embryo after heat shock [2].

Heat shock did induce expression of some heat shock protein and antioxidant genes. As determined in the 3’DGE analysis, heat shock increased expression of *HSPB11* significantly and tended to increase expression of *HSPA1A* and *HSPB1.* Also, four genes regulated by NRF2, a transcription factor activated by oxidative stress [42], were increased by heat shock *(AKR7A2, CBR1, GSTA4,* and *MAP2K5)* as was another gene involved in glutathione metabolism (*GGH*). The experiment using real-time PCR was performed to determine if effects of heat shock on expression of selected heat shock protein and antioxidant genes depended on the duration of heat shock. Results indicated that responses to heat shock were similar after 2, 4 and 8 h of exposure to elevated temperature, Moreover, the apparent increase in expression of *HSPA1A* detected by 3’DGE was repeatable and significant, as would be expected from previous papers [2,43], and there was a tendency for expression of *HSP90AA1* to increase slightly after heat shock. The increase was small and of a magnitude similar to that seen in the 3’DGE experiment (although the difference was non-significant). In an earlier experiment, there was no increase in steady-state amounts of *HSP90AA1* after 24 h of heat shock in one-cell or Day 5 embryos [2]. There was also no effect of heat shock of any duration on expression of *CAT* or *SOD1*, in agreement with the results of 3’DGE and an earlier experiment in which embryos were exposed to 24 h of heat shock [2].

However, heat shock did not alter expression of 64 other genes that encoded heat shock proteins, heat shock transcription factors or heat shock protein binding proteins or expression of 42 other genes involved in antioxidant defense. Analysis of the literature and the results from the present study provide clues as to why the induction of expression of heat shock protein and antioxidant genes was so muted. The lack of a large-scale heat shock protein response could reflect efficient clearance of denatured protein. The most striking change in response to heat shock in the current study was a change in expression of genes encoding for proteins that bind to UBC. This protein is part of the ubiquitination pathway that is critical for removal of damaged protein including in cells damaged by heat shock [46]. Changes in expression of genes involved in the ubiquitin pathway could be reflective of perturbation of the ubiquitin-proteasome pathway by heat shock. Removal of damaged proteins by this pathway could conceivably reduce the signal for activation of HSF1, the transcription factor controlling expression of heat shock protein genes, because this protein is activated as a result of increased accumulation of denatured protein [39]. The idea that embryos that have developed thermotolerance are less likely to accumulate denatured proteins than embryos at earlier stages is consistent with earlier results in mouse embryos, where a more severe heat shock was required to induce HSP70 synthesis in blastocysts than in eight-cell embryos [47].

The lack of broad increase in genes involved in antioxidant defense probably reflects the inhibition of free radical accumulation in response to heat shock for embryos at this stage of development. Previous results indicate that heat shock did not increase free radical production at Day 4 or 6 after fertilization (16-cell through morula stage of development) although it did when embryos were at Days 0–2 of development (one-cell to early eight-cell stage) [5]. The most likely cause is increased accumulation of intracellular antioxidants. Indeed, one such antioxidant, glutathione, increases in amount by the 9–16 cell stage [18].

One mechanism contributing to the thermotolerance of the preimplantation embryo is the acquisition of the capacity for apoptosis at the 8–16 cell stage [48]. At physiological temperatures, heat shock causes apoptosis in about 10-20% of the blastomeres [49-51]. Inhibition of apoptosis through administration of caspase 3 inhibitors exacerbates the effects of heat shock on development [51,52]. In the present study, effects of heat shock on genes involved in apoptosis were inconsistent. Three genes that are associated with activation of apoptosis were increased by heat shock: *BOK*[53], *NGFR*[54] and *STK4,* which activated apoptosis through a p53-dependent mechanism [55]. However, another gene that inhibits apoptosis, *SERINC3*[56] was increased by heat shock and the pro-apoptotic gene, *PDIA3*[57], was decreased by heat shock. The possible involvement of the ubiquitin system in clearance of proteins damaged by heat shock, as indicated by the large number of genes regulated by heat shock that bind UBC, may also have implications for apoptosis since ubiquitination plays a role in activation of apoptosis responses [58].

There were few genes involved in developmental processes whose expression was affected by heat shock. This is not surprising because, as seen previously [2,5,6], the development of the morula-stage embryo to the blastocyst stage was unaffected by heat shock. It is likely that adjustments to cellular function that limited the heat shock protein and antioxidant gene response to heat shock also allowed development to continue normally after exposure to 40°C. It may be, however, that some changes in gene expression induced by heat shock compromise ability of the blastocyst to continue development in later embryonic or fetal life. In one study in which Day 4 embryos were exposed to a more severe heat shock than used here (41°C for 12 h), pregnancy rate of the blastocysts formed after heat shock was lower after transfer into recipient females than for blastocysts formed from control embryos [59]. Among the pathways affected by heat shock that could potentially compromise subsequent development was the WNT signaling pathway, where heat shock increased expression of *AXIN1* and *LEF1* and decreased expression of *CUL1* and *PPP2A*. WNT signaling regulates cell proliferation, cell fate decision and stem cell maintenance [60]. *LEF1* is involved in trophectoderm differentiation [37], and *CUL1* is required for development in the mouse [38]. Heat shock also caused changes in expression of genes regulating mucopolysaccharide synthesis. One mucopolysaccharide, hyaluronan, has been reported to increase competence of morula-stage and early blastocyst-stage bovine embryos to establish pregnancy after transfer into recipients [61]. Heparan sulfate proteoglycans also regulate WNT availability by binding to secreted WNTs [62].

## Conclusions

In conclusion, changes in the transcriptome of the heat-shocked bovine morula indicate that the embryo is largely resistant to effects of heat shock. As a result, activation of transcription of genes involved in thermal protection is muted and there is little indication of disruption in gene networks involved in embryonic development. It is likely that the increased resistance of morula-stage embryos to heat shock as compared to embryos at earlier stages of development is due in part to development of mechanisms to prevent accumulation of denatured proteins and free radical damage.

## Abbreviations

3’DGE: 3’ tag digital gene expression; DAVID: Database for Annotation, Visualization and Integrated Discovery; DPBS: Dulbecco’s phosphate-buffered salaine; hpi: Hours post-insemination; ICM: Inner cell mass; IPA: Ingenuity Pathways Analysis; PCR: Polymerase chain reaction; PVP: Polyvinylpyrrolidone (3’DGE); TE: Trophectoderm.

## Competing interests

The authors state that they have no competing interests.

## Authors’ contributions

Conceived and designed experiments: MS, PJH. Carried out experiments: MS, KBD, JB, MO, SS. Analyzed data: MS, JY. Wrote the initial drafts of the paper: MS, SS, JY, PJH. All authors read and approved the final manuscript.

## Supplementary Material

Additional file 1: Table S1 Genes whose expression was differentially expressed by heat shock in bovine morula. Shown (from left to right) are the ENSEMBL gene identification number, the number of counts for each of three control samples (C5, C6, C14) and three heat-shock samples (H5, H6, H14), the average count, the mean counts for control (MeanC) and heat shock (MeanH), the fold change (heat shock/control), the standard error of the difference, the p value, gene name and gene description. Genes that are upregulated by heat shock are shaded light red while those that are downregulated are shaded green. Click here for file
